# Is mTOR inhibition a systemic treatment for tuberous sclerosis?

**DOI:** 10.1186/1824-7288-39-57

**Published:** 2013-09-17

**Authors:** Romina Moavero, Antonella Coniglio, Francesco Garaci, Paolo Curatolo

**Affiliations:** 1 Systems Medicine Department, Child Neurology and Psychiatry Unit, Tor Vergata University Hospital of Rome, Via Montpellier 1, 00133, Rome, Italy; 2 Department of Diagnostic Imaging and Interventional Radiology, Tor Vergata University Hospital, Via Montpellier 1, 00133, Rome, Italy

**Keywords:** Tuberous sclerosis, Everolimus, mTOR inhibitors, Epilepsy, Treatment, SEGA, Renal angiomyolipomas

## Abstract

Tuberous sclerosis complex (TSC) is a genetic multisystem disorder characterized by the development of hamartomas in several organs. Mutations in the TSC1 and TSC2 tumor suppressor genes determin overactivation of the mammalian target of rapamycin (mTOR) signaling pathway and subsequent abnormalities in numerous cell processes. As a result, mTOR inhibitors such as sirolimus and everolimus have the potential to provide targeted therapy for TSC patients. Everolimus has been recently approved as a pharmacotherapy option for TSC patients with subependymal giant-cell astrocytomas (SEGAs) or renal angiomyolipomas (AMLs). However, clinical evidence suggests that this treatment can benefit other TSC-associated disease manifestations, such as skin manifestations, pulmonary lymphangioleiomyomatosis, cardiac rhabdomyomas, and epilepsy. Therefore, the positive effects that mTOR inhibition have on a wide variety of TSC disease manifestations make this a potential systemic treatment option for this genetic multifaceted disorder.

## Introduction

Tuberous sclerosis complex (TSC) is a variably expressed autosomal dominant genetic disorder characterized by the presence of benign, non-invasive, tumor-like lesions (hamartomas) in the brain, heart, skin, kidney, lung, and liver [1]. This disorder has an estimated birth incidence of approximately 1 in 6000 [2]. Central Nervous System is almost invariably affected in TSC (85–90% of children and adolescents), causing disabling neurological manifestations, including epilepsy (up to 90% of patients with TSC), subependymal nodules (SENs; 90–100%), subependymal giant cell astrocytomas (SEGAs; 5–20%), and mental delay (44–64%) [1]. Other brain manifestations include widespread microstructural white matter abnormalities [3]. Non neurologic manifestations include hypomelanotic macules and facial angiofibromas, renal cysts and/or angiomylipomas, pulmonary lymphangioleiomyomatosis, cardiac rhabdomyomas, retinal hamartomas and hepatic angiomas [1]. In TSC, mutations in one of the two tumor suppressor genes, TSC1 (encoding hamartin) or TSC2 (encoding tuberin), are found in more than 85% of cases [4]. Hamartin and tuberin are involved in the regulation of cell proliferation and differentiation, forming a physical and functional complex that activates guanosine triphosphatase (GTPase), keeping the protein Ras homolog enriched in brain protein (RHEB) inactive in order to inhibit the mammalian target of rapamycin (mTOR) pathway. The mTOR pathway is responsible for protein and lipid biosynthesis and growth factor-related cell cycle progression. Under normal circumstances, hamartin and tuberin are activated ivia biosynthetic processes mediated by the mTORC1 complex, which includes mTOR, raptor (mTOR regulatory-associated protein of mTOR), mLST8, and PRAS40 (proline-rich Akt substrate 40) [5]. Therefore, TSC1 or TSC2 mutations give rise to hyperactivation of the mTOR pathway, resulting in a downstream kinase signaling cascade that can lead to abnormalities in numerous cell processes, including cell cycle progression, transcription, translation, and metabolic control [5]. Progress in understanding the molecular pathophysiology of TSC and the crucial role of mTOR hyperactivation in determining most of the clinical features in TSC paved the way to the development of new therapeutic strategies involving mTOR inhibitors. This review will focus on the current role of mTOR inhibitors in systemic treatment of different TSC related manifestations.

### Neurologic manifestations of TSC

#### Subependymal giant cell astrocytomas

Subependymal giant cell astrocytomas (SEGAs) are slow-growing tumors of mixed cellular lineage, occurring in about 5–20% of TSC patients, and represent a significant cause of morbidity and mortality because of the risk of sudden death from acute hydrocephalus [6,7].

mTOR inhibitors’ efficacy in determining a reduction of SEGA volume is now well established, so that Everolimus has been the first drug specifically licensed in the USA and Europe for the treatment of TSC patients aged ≥3 years with TSC-related SEGA who require therapeutic intervention, but are not candidates for curative surgical resection.

The evidence of this efficacy comes from phase I/II open-label trial as well as from an international phase III study [8,9]. EXIST-1 (Examining everolimus In a Study of TSC) was a phase III international, multicenter, double-blind, randomized, placebo-controlled trial that evaluated the efficacy and safety of everolimus in 117 patients with SEGA associated with TSC. The primary endpoint of EXIST-1 was the proportion of patients with a SEGA response (confirmed by MRI 8–12 weeks after the response), defined as a reduction from baseline of ≥50% in the sum volumes of all target SEGA lesions, non-worsening of non-target SEGA lesions, no new SEGA lesions ≥1 cm, and no new/worsening hydrocephalus. Everolimus was associated with a significantly greater overall SEGA response rate, compared with placebo (35% vs. 0%; p < 0.0001); this benefit was consistent across all patient subgroups analyzed [8]. The median time-to-SEGA progression was not reached, but the estimated progression-free rate at 6 months was significantly higher with everolimus (100% vs. 86%; p = 0.0002).

**Figure 1:**
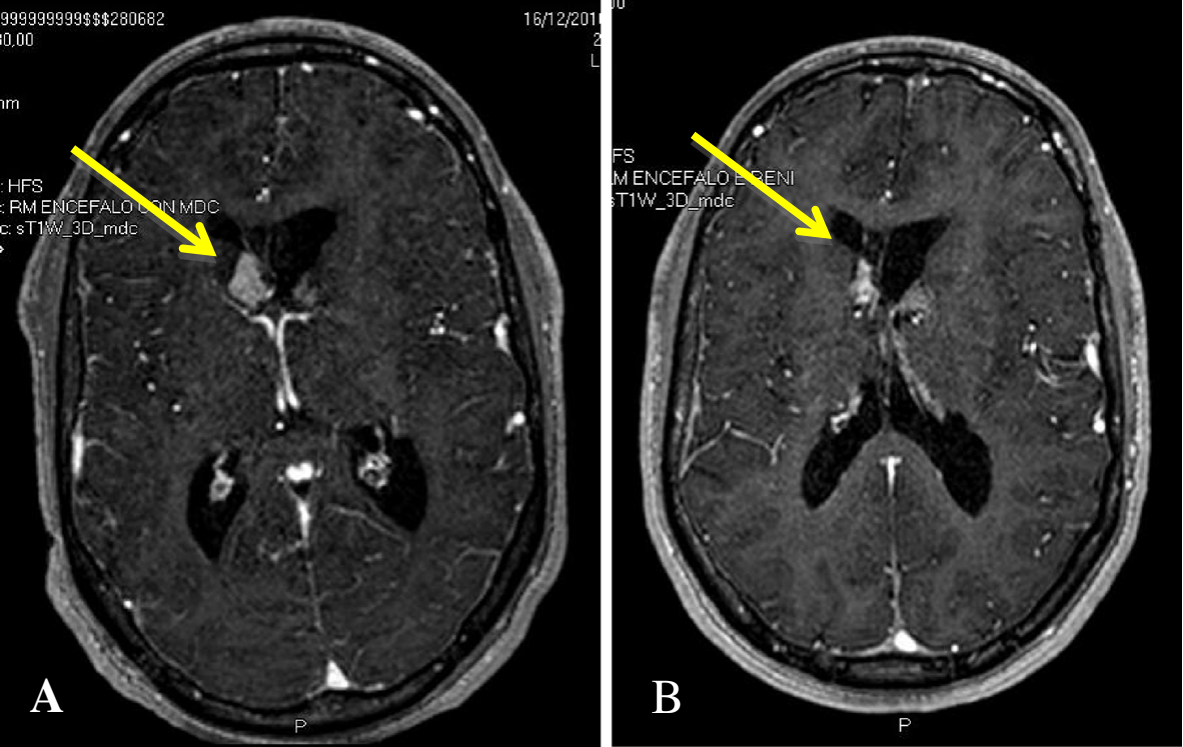
**SEGA volume reduction during Everolimus treatment.** A boy with a TSC2 mutation showed progressively growing SEGAs that caused a first acute hydrocephalus when he was 17 years old. He therefore underwent a partial resection of the lesion, with hydrocephalus resolution. However, two years later, the contralateral SEGAs determined a new episode of hydrocephalus and he underwent surgery once again. However, both lesions presented a slow regrowth after surgeries, and figure **(A)** shows their aspect when the boy was 24 years of age. He later presented a new episode of subacute hydrocephalus, which required an external derivation. He subsequently started Everolimus treatment, with a partial response (total lesion volume 46% of baseline) of SEGAs after 12 months of treatment **(B)**.

In our experience with Everolimus, SEGA volume reduction was greater in the first 3 months of treatment, but SEGAs usually continued to respond until at least 12–18 months of continuative treatment. Furthermore, in patients with a pre-existing hydrocephalus, ventricles’ enlargement appeared to significantly decrease even before a significant reduction of SEGA volume. In particular, in our series, one patient began his treatment with Everolimus soon after an episode of subacute hydrocephalus treated with an external drainage, and although SEGA volume shrinked to less than 50% only after 12 months of treatment, ventricles’ enlargement was significantly decreased after the first three months of pharmacotherapy (Figure 1).

#### Epilepsy

Epilepsy associated with tuberous sclerosis generally begins during the first year of life and, in most patients, in the first few months. Focal seizures may precede, coexist with, or evolve into infantile spasms [1]. Animal models showed that mTOR inhibitors proved to have antiepileptic and even antiepileptogenic effect, decreasing seizures when started after epilepsy onset of seizures, or preventing the development of epilepsy when initiated prior to the onset of seizures [10]. There is some evidence showing that rapamycin might be a disease modifier agent in epileptic spasms, even not specifically related to TSC, but it doesn’t seem to have the same efficacy on other seizure types [11]. Different models show that beneficial effects on seizures are lost when treatment is withdrawn, suggesting that mTOR inhibitors are “epileptostatic” in only stalling epilepsy progression during treatment [12].

Clinical studies of rapamycin in human epilepsy are limited, but suggest that mTOR inhibitors at least have antiseizure effects in tuberous sclerosis patients. Further studies are needed to assess the full potential of mTOR inhibitors for epilepsy treatment. Up to now there are only few clinical data reporting about efficacy and safety of mTOR inhibitors in epilepsy secondary to TSC. Most of these data come from clinical trials investigating the efficacy of Everolimus on SEGAs, or from anecdotical case reports. The EXIST-1 trial failed to demonstrate a reduction in seizure frequency after 6 months of Everolimus treatment [8]. On the other hand, single case reports seem to indicate some efficacy in reducing seizure frequency in TSC patients receving mTOR inhibitors [13,14].

**Figure 2:**
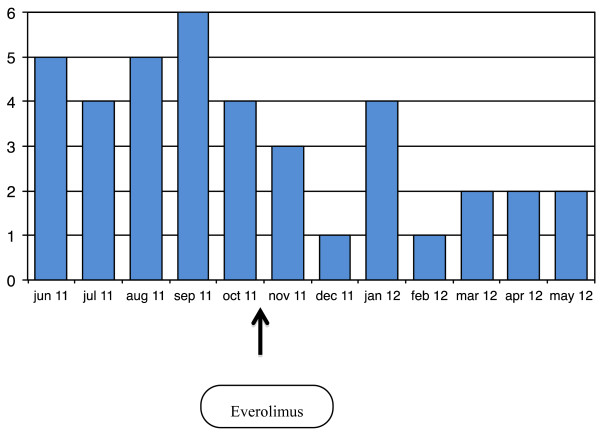
**Monthly seizure frequency in a TSC girl.** A girl with a TSC2 mutation presented refractory seizures since the age of 7 years. When she was 20 years old she presented mainly secondarily generalized tonic and tonic-clonic seizures. The image shows her seizure frequency per month before and during Everolimus treatment. With everolimus seizure frequency decreased, generalized seizures stopped, and she continued to have only focal onset seizures of short duration, without drop attacks nor falling to the ground.

In our clinical experience of TSC epileptic patients treated with Everolimus for SEGA and/or AMLs, epileptic seizures appeared to decrease both in frequency and in severity (Figure 2). Furthermore, seizures tend to become less dangerous for the patient, thus determining a minor impact on the patient’s quality of life. However, after the discontinuation of treatment seizures promptly appeared again with the same frequency as before Everolimus. In the clinical practice caution should be used when Everolimus is added to antiepileptic drugs such as especially carbamazepine, topiramate, barbiturates and phenytoin; clinicians should be aware of the pharmacokynetic and pharmacodynamic interactions between these different drugs, as well as of the possible adverse events.

#### Mental delay and autism

TSC associated neurocognitive manifestations are quite frequent in TSC, and highly variable in their expression. Cognitive impairment is present in about half of TSC patients, with 30% being profoundly impaired [1]. Most important variables associated with poor cognitive outcome and autism spectrum disorder include a history of refractory seizures, mutations of TSC2, and the presence of cortical tubers in certain regions [15], but age at seizure onset appears to be one of the major contributor to cognitive function [16]. About 70–75% of patients experiencing seizures in the first year of life, including infantile spasms, could present a later cognitive impairment [17], however an early and effective treatment soon after seizure onset can significantly ameliorate the final outcome, although it is not able to totally reverse TSC- associated cognitive impairment [18,19]. Evidence on animal models showed that mTOR signalling plays a crucial role in different social behaviours as well as in learning deficit [20], and a brief treatment with rapamycin rescued not only synaptic plasticity but also behavioral deficits [21]. Furthermore the treatment both before and after early seizures with mTOR inhibitors was able to decrease both seizure susceptibility and later autistic-like behaviours [22], thus supporting the hypothesis that mTOR inhibitors could be useful for the pharmacological treatment of TSC associated autism spectrum disorders. Recent clinical xevidence also showed that treatment with mTOR inhibitors appeared to be able to decrease white matter abnormalities, even if the exact clinical significance of this finding is still unknown (Tillema et al., 2012). Clinical trials to examine the effects of mTORC1 inhibitors on neurocognitive function, autistic phenotypes and epilepsy are currently underway (ClinicalTrials.gov; NCT01289912, NCT01070316).

### Non-neurologic manifestations of TSC

#### Renal AMLs

Renal complications are the most frequent cause of tuberous-sclerosis-related death [6]. Multiple, bilateral angiomyolipomas are found in about 70–90% of adult patients [1]. Their frequency is lower in children than in adults, but up to 16% of patients below the age of 2 years can be affected [23]. These tumours, consisting of abnormal blood vessels, smooth muscle, and adipose tissue, tend to grow and spontaneous bleeding is the most common complication in patients with tumours larger than 4 cm in diameter [1].

Different clinical trials have been performed in order to establish the efficacy and safety of mTOR inhibitors in reducing AML size. Two different studies administering sirolimus reported a reduction in tumor volume of more than 50%, but a regrowth after treatment cessation [24,25]. Furthermore, also in this case an international, multicenter, double-blind, randomized, placebo-controlled study has been carried on, assessing the efficacy and safety of everolimus in 118 patients with AML associated with TSC or sporadic lymphangioleyomyomatosis (LAM). The primary efficacy endpoint for EXIST-2 was the proportion of patients who achieved a best overall AML response (confirmed by kidney computed tomography/MRI 8–12 weeks after the response), which was defined as a reduction from baseline of ≥50% in the sum of volumes of all target AML lesions, no new lesions ≥1 cm in the longest diameter, no kidney volume increase of >20% from nadir, and no AML-related bleeding grade ≥2 (defined by the National Cancer Institute Common Terminology Criteria for Adverse Events, version 3.0). Everolimus was associated with a significantly greater AML response rate, compared with placebo (41.8% vs. 0%; p < 0.0001); this benefit was consistent across all patient subgroups analyzed. The median time-to-AML progression was 11.4 months in the placebo group, but was not reached in the everolimus treatment group (hazard ratio 0.08; 95% CI 0.02-0.37; p < 0.0001); estimated 6-month progression-free rates of 98.4% and 83.4% were reported in the everolimus and placebo groups, respectively [26].

**Figure 3:**
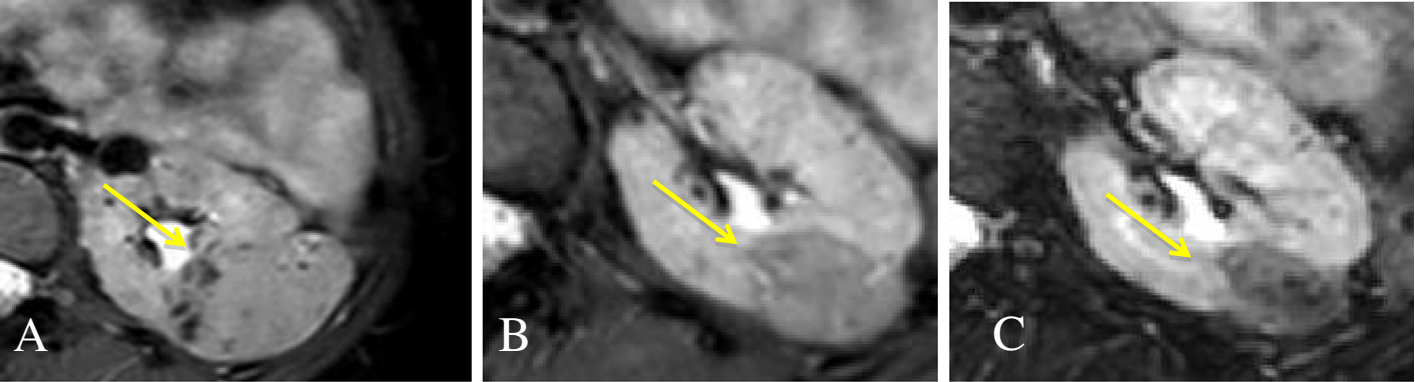
**Progressive reduction of renal AMLs.** A girl with a TSC2 mutation presented renal AMLs since childhood, with no impairment of renal function but with a progressivre growth both in number and in size. When she was 23 years old Everolimus was started (**A**, baseline), with a progressive reduction in the total volume of lesions. Figure **B** is the MRI after 12 weeks of treatment (76% of volume reduction), while figure **C** is after 144 weeks of treatment with Everolimus (85% of volume reduction).

In our series AMLs responded even better than SEGAs. When the patient is a responder, already in the first three months a reduction of more than 50% of the baseline volume is observed. In our experience with long-term treatment, we observed a continuous progression of volume decrease even after 2 or 3 years of continuative treatment, in one case with the total lesion volume decreasing up to 13% of the baseline (Figure 3).

#### Dermatologic manifestations

Bilateral facial angiofibromas are hamartomatous nodules of vascular and connective tissue, with a butterfly pattern over the malar eminences and nasal labial folds of the face [1]. Their frequency is about 80% in children with tuberous sclerosis who are older than 5 years of age [27]. Dermatologic manifestations response to mTOR inhibitors has been extensively investigated in recent years. Most of the studies have been specifically performed with topical formulation of sirolimus, but some indirect data also come from the systemic administration of sirolimus/everolimus in patients treated for AML or SEGAs [8,24,26].

Most of the actual experience up to now is with facial angiofibromas, which appears to present a good response to topical administration of mTOR inhibitors, even if a standardized protocol of use is still lacking [28-31]. Topical rapamycin formulation has also been tested in hypomelanotic macules, showing some efficacy even if up to now there are only two reported cases [32].

**Figure 4:**
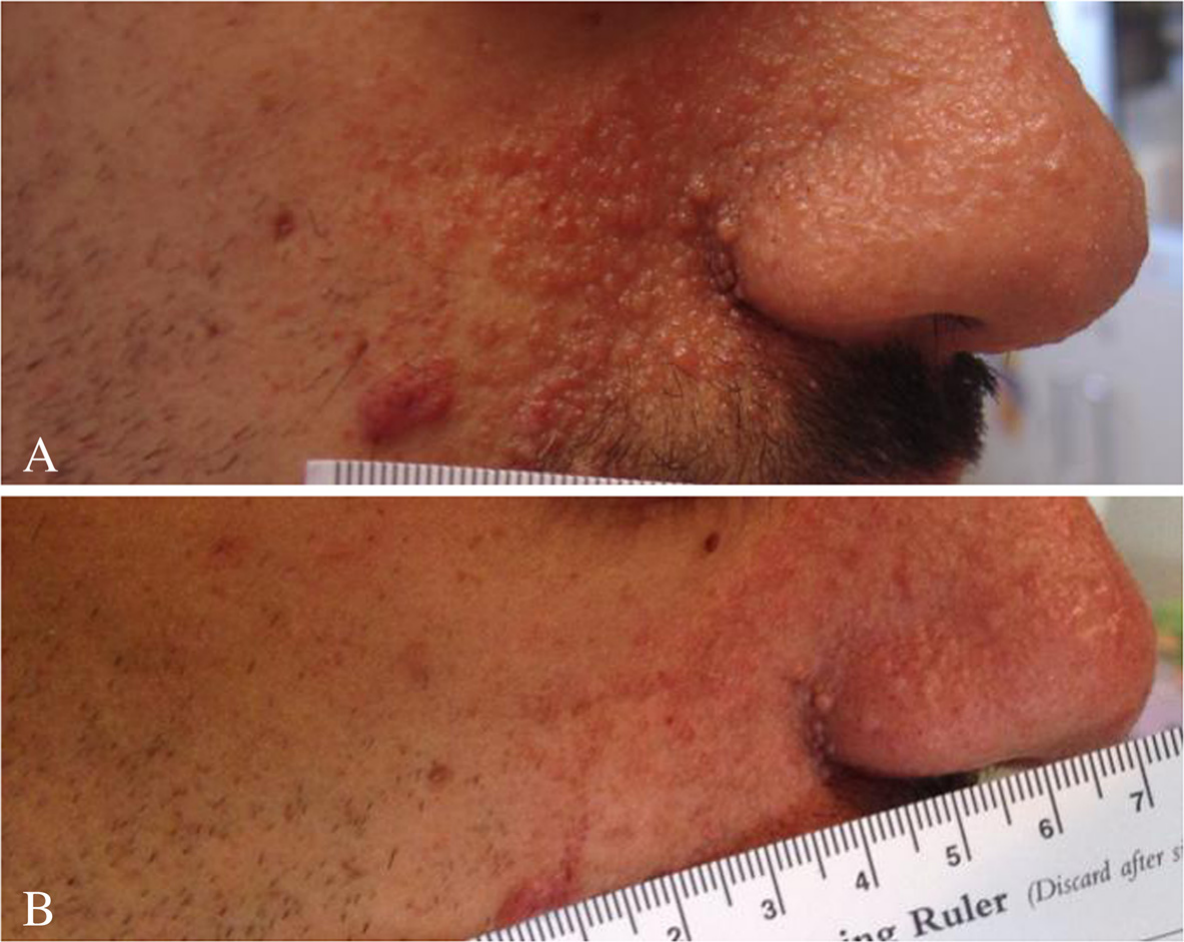
**Facial angiofibroma before and during Everolimus treatment.** Facial angiofibroma of the same boy presented in Figure 1 at baseline **(A)** and after 84 weeks of treatment with Everolimus **(B)**.

Some of the patients we treated for SEGAs and/or AMLs also presented facial angiofibromas. During everolimus treatment these skin lesions tended to clear up, and became more plan already after 2–4 weeks of treatment (Figure 4).

#### Cardiac rhabdomyomas

Cardiac rhabdomyomas are the main feature of the disease in the fetus and newborn baby. 96% of infants with cardiac rhabdomyomas will ultimately be diagnosed with tuberous sclerosis. Although patients typically have several, these tumours are rarely symptomatic. Nonetheless, they can manifest prenatally as arrhythmia, non-immune hydrops, or death [1]. Cardiac rhabdomyomas are the main feature of the disease in the fetus and newborn baby. Lesions may be completely asymptomatic and usually recede over time, with complete regression in childhood [1,33]. However, before their regression, cardiac rhabdomyomas may cause clinically significant arrhythmias, ventricular inflow and outflow obstruction, and congestive heart failure. For these patients, medication is needed to treat arrhythmias and heart failure, and surgery may be required to relieve obstruction [1,34].

A recent case report described the regression of a cardiac rhabdomyoma in a 7 year old boy treated with Everolimus for SEGA [34]. Even if a natural regression can not be excluded, the rapidity of the regression may suggest that Everolimus could have played a significant role. Further data are necessary to understand if TSC related cardiac manifestations could be a target for mTOR inhibiton.

#### LAM

Pulmonary lymphangiomyomatosis predominantly affects fertile women and can be progressive and with a poor prognosis [1]. Positive data regarding mTOR inhibitors efficacy in pulmonary LAM come both from case series and from specific randomized trials, even if these were not initially designed for TSC patients [35-37]. A phase III, randomized and double-blind placebo controlled trial have been performed investigating the efficacy and safety of sirolimus in lymphangioleyomiomatosis in adult patients with and without TSC. This study demonstrated that at the end of the treatment period, FEV1 stabilized or improved in 46% of patients on sirolimus, but when the drug was stopped, lung function decline resumed [35]. Sirolimus treated group also showed an improvement in forced vital capacity (FVC) and in measures of functional performance and quality of life, but not in exercise tolerance.

Furthermore, clinical assessing sirolimus/everolimus treatment in TSC-related AML showed a slight improvement, or at least a stabilization, in lung function in patients affected by LAM [24-26].

### Current role in the clinical practice

mTOR inhibition is a novel therapeutic approach that can address multiple aspects of the TSC disease at the same time. In TSC the major causes of mortality are SEGA-related hydrocephalus, status epilepticus, hemorrhage due to renal AMLs, and LAM progression [1,6]. Everolimus could have the potential to provide targeted treatment for the entire spectrum of TSC-related manifestations, reducing the risk of life-threatening complications. However, drug therapy can not replace at all surgery nor other therapeutic options, and every clinical choice should be carefully tailored on the single patient taking into account the risks and benefits of current treatment options. mTOR inhibitors may be recommended when asymptomatic SEGA is observed to be growing in two subsequent MRI evaluations, or even as initial treatment to facilitate subsequent surgery in patients with bilateral lesions [7]. The pharmacotherapy with mTOR inhibitors may replace surgery when SEGAs present an atypical localization, exhibit aggressive growth, or after a re-growth in case the second surgery is associated with a higher risk of complications. Furthermore, the concomitant presence of a growing AML and of intractable seizures should be taken into account when clinicians are making decisions between the two treatment options for an individual patient [38].

In general, mTOR inhibitors are well tolerated. The majority of adverse events are linked to the immunosuppressive action of this drug class, and include aphthous ulcers, fever, fatigue, rash, mucositis, loss of appetite, gastrointestinal effects such as diarrhea and nausea, arthralgias, thrombocytopenia, and effects on lipid metabolism [8]. In most cases, these AEs are self-limiting and can be managed by dose reductions or specific symptomatic therapy, discontinuation of treatment is not usually necessary [39]. Potentially serious AEs may include upper respiratory tract infections as well as non-infective pneumonitis and dramatic elevations in serum cholesterol and lipoprotein levels, which may require dietary adjustment or the use of cholesterol-lowering medication [8]. Apparently a definite indication on the most safe therapeutic dosage of Everolimus is still lacking. Most of the recent studies used it at the dosage able to determine a serum concentration of 3–5 up to 15 ng/ml [8,26], but more clinical experience is needed to clarify this issue, especially in early childhood.

One of the still open clinical questions regards the optimal timing for starting treatment with Everolimus. When considering the best treatment option for young children, the potential adverse events of a long-term pharmacotherapy with these immunosuppressant agents must be always taken into account. The positive effects that mTOR inhibition have on a wide variety of TSC disease manifestations make this a potential systemic treatment option for this genetic multifaceted disorder. Further studies are needed to clarify the long-term clinical efficacy, the optimal dosage regimen in order to better define the role of mTOR inhibitors in the treatment of TSC related manifestations.

## Consent

Written informed consent was obtained from the patient’s guardian/parent/next of kin for the publication of this report and any accompanying images.

## Abbreviations

AML: Angiomyolipomas; FEV1: Forced expiratory volume in the 1st second; FVC: Forced vital capacity; GTPase: Guanosine triphosphatase; LAM: Lymphangioleyomyomatosis; MRI: Magnetic resonance imaging; mTOR: Mammalian target of rapamycin; mTORC1: mTOR complex1; PRAS40: Proline-rich Akt substrate 40; RHEB: Ras homolog enriched in brain protein; SEGA: Subependymal giant cell astrocytoma; SEN: Subependymal nodules; TSC: Tuberous sclerosis complex.

## Competing interest

The authors declare that they have no conflict of interest to declare.

## Authors’ contributions

RM wrote the preliminary draft; PC initially designed the paper. RM and PC performed the review of the literature. FGG performed and analyzed the imaging assessments. AC performed the clinical follow up of patients. All authors read and approved the final manuscript.
